# Concomitant Potentially Contagious Factors Detected in Poland and Regarding *Acanthamoeba* Strains, Etiological Agents of Keratitis in Humans

**DOI:** 10.3390/microorganisms12122445

**Published:** 2024-11-28

**Authors:** Lidia Chomicz, Jacek P. Szaflik, Agnieszka Kuligowska, David Bruce Conn, Wanda Baltaza, Beata Szostakowska, Paweł J. Zawadzki, Monika Dybicz, Anna Machalińska, Konrad Perkowski, Anna Bajer, Jerzy Szaflik

**Affiliations:** 1 Department of Medical Biology, Medical University of Warsaw, 00-575 Warsaw, Poland; 2 Department of Ophthalmology, Independent Public Clinical Ophthalmology Hospital, Medical University of Warsaw, 00-576 Warsaw, Poland; okovisus@gmail.com; 3 First Department of Ophthalmology, Pomeranian Medical University, 70-111 Szczecin, Poland; anna.machalinska@pum.edu.pl; 4 Department of Invertebrate Zoology, Museum of Comparative Zoology, Harvard University, Cambridge, MA 02138, USA; bconn@berry.edu; 5 One Health Center, Berry College, School of Mathematical and Natural Sciences, Mount Berry, GA 30149, USA; 6 Department of Public Health, Medical University of Warsaw, 02-097 Warsaw, Poland; wanda.baltaza@wum.edu.pl; 7 Department of Tropical Parasitology, Faculty of Health Sciences, Medical University of Gdansk, 80-210 Gdansk, Poland; beata.szostakowska@gumed.edu.pl; 8 Clinic of Cranio-Maxillo-Facial and Oral Surgery and Implantology, Medical University of Warsaw, 02-005 Warsaw, Poland; pawel.j.zawadzki@wum.edu.pl; 9 Department of General Biology and Parasitology, Medical University of Warsaw, 02-004 Warsaw, Poland; monika.dybicz@wum.edu.pl; 10 Department of Orthodontics, Medical University of Warsaw, 02-006 Warsaw, Poland; konrad.perkowski@wum.edu.pl; 11 Department of Eco-Epidemiology of Parasitic Diseases, Institute of Developmental Biology and Biomedical Sciences, University of Warsaw, 02-096 Warsaw, Poland; a.bajer2@uw.edu.pl; 12 Laser Eye Microsurgery Centre Clinic of Prof. Jerzy Szaflik, 00-215 Warsaw, Poland; jerzy@szaflik.pl

**Keywords:** microbiota, infectious strains, concomitant factors, corneal isolates, confocal microscopy, in vitro methods, cellular and molecular diagnostics, *Acanthamoeba* keratitis, co-occurring infections, non-invasive in vivo methods

## Abstract

Background: Diseases in humans caused by amphizoic amoebae that can result in visual impairment and even blindness, have recently been identified more frequently worldwide. Etiologically complex incidents of keratitis, including those connected with *Acanthamoeba* strains detected in Poland, were evaluated in this study. Methods: Corneal samples from cases resistant to antimicrobial therapy assessed for epidemiological, microbiological and parasitological aspects were investigated by phase-contrast microscope, slit lamp and by confocal microscopy. In vitro techniques were applied for detection of bacteria and fungi, and corneal isolates cultured under axenic condition using BSC medium—for detection of *Acanthamoeba* spp.; molecular techniques were applied for amoeba species identification. Results: Most etiologically complicated keratitis cases, detected in ~84% of incidents, was due to exposure of contact lenses to tap water or pool water; trophozoites and cysts of *Acanthamoeba*, concomitant bacteriae, e.g., *Pseudomonas aeruginosa*, fungi and microfilariae were identified in contact lens users. Conclusions: In samples from contact lens wearers where microbial keratitis is identified along with some connection with the patient’s exposure to contaminated water environments, a risk of *Acanthamoeba* spp. infections should be considered. Understanding the complicated relationship between *Acanthamoeba* spp., co-occurring pathogens including associated endosymbionts is needed. In vivo confocal microscopy and in vitro cultivation were necessary to identify potentially contagious concomitant factors affecting the complex course of the keratitis.

## 1. Introduction

Free-living amoebae of the genus *Acanthamoeba* exist in two morphological forms: as metabolic active trophozoites with spine-like acanthopodia, and as double-walled dormant cysts that can develop after trophozoite growth phase and under harsh conditions.

The amoebae are common in natural and man-made habitats worldwide. They occur in air, water, soil and dust; they are found in/on vegetables, animals and humans.

The protozoans have been detected in air conditioning and in the municipal sewage systems, are present on the surfaces of various equipment and accessories in the health facilities, e.g., dental irrigation unit and dialyzers. The amoebae are often found in various aquatic habitats such as in tap water, chlorinated swimming pools and sea water [1,2,3,4,5,6,7,8,9,10,11,12,13,14,15,16,17,18,19].

*Acanthamoeba* spp., as the exozoic forms, complete their life cycles in the external environment; however, it is known that under predisposing circumstances, some strains of *Acanthamoeba* are able to enter human bodies and exist as the endozoic forms, causing pathogenic effects [1,2,3,4,5,6,7,8]. Thus, the amoebae are believed to be amphizoic organisms that can exist as free-living forms and as facultative parasites of human tissues and systems.

Parasitic predispositions of *Acanthamoeba* species can be expressed as a serious threat for human health for persons undergoing immunosuppressive therapy, and HIV/AIDS patients. Some *Acanthamoeba* strains are causative agents of the granulomatous amoebic encephalitis (GAE), which is usually a fatal disease developing in immunocompromised individuals; the amoebae have been detected in human organs, on the surface of skin, in paranasal sinuses and in the lungs [3,4,5,6].

Simultaneously, pathogenic *Acanthamoeba* strains present a serious risk for human health as the causative agents of *Acanthamoeba* keratitis (AK) [1,2,3,4,5]. The vision-threatening, potentially blinding corneal infection is presented as a non-opportunistic disease that affects immune-competent persons.

Currently, the growing threat to public health generated by amphizoic amoebae, the causative agent of AK, has been diagnosed with increasing frequency in Poland [6,9,10,11,12,13,14].

Poor hygiene during contact lens (CL) use is considered highest risk factor for *Acanthamoeba* keratitis. It is emphasized that eye infections caused by *Acanthamoeba* strains have been found more often as the popularity of contact lens use has been increasing.

The amoebae can enter human bodies also without pathogenic consequences. It has been shown by the serological, biochemical and molecular methods that people may be exposed to both the pathogenic and the non-pathogenic *Acanthamoeba* strains. Evidence from various regions confirmed that humans are frequently exposed to the amoebae: 50–100% of healthy populations with no history of amoebic infections had the specific anti-*Acanthamoeba* antibodies [3,6,7,8].

*Acanthamoeba* keratitis may include non-specific symptoms that are similar to those observed in the course of viral, bacterial or fungal keratitis. Free-living amoebae that feed on various microorganisms may play an important role in the environmental transmission of developmental forms of bacteria and fungi, which are pathogenic for humans, as the causative agents of microbial keratitis [3,9,12].

The *Acanthamoeba* strains can also disperse different microbiota that are able to survive within amoebae as endosymbionts [15,16,17,18,19,20,21,22,23,24,25,26,27,28].

Clinically or environmentally related microbiota, etiological factors of eye diseases, are found in cases of co-infections with amphizoic amoebae [3,6,9,17,18,19,20,22,29,30].

The interdisciplinary study was performed to determine and assess the concomitant potentially contagious factors and regarding *Acanthamoeba* strains detected in Poland, which are causative agents of keratitis in humans.

## 2. Materials and Methods

Corneal samples originating from complicated cases of keratitis detected in men and women, aged 22–45 years, were examined in our departments in terms of causative factors of visual impairment.

Earlier, the incidents were treated with different medications in several ophthalmic units, without success. An appearance of a resistance to initially applied antimicrobial therapy has been taken into account as the important criterion for inclusion of such cases into our study.

The time from symptom onset to detection of etiological agents of keratitis was determined, and diagnostic verification was performed for these incidents. Corneal material was examined to evaluate factors that could cause pathogenic changes in the eyes, posing a serious risk for human health.

The study was performed in accordance with the tenets of the Declaration of Helsinki. The data from tests obtained with use of the clinical and laboratory methods have been assessed.

**The clinical assessment**

Clinical pictures of affected eyes appearing in keratitis with different intensity—e.g., strong photophobia, redness, pain, excessive lacrimation, reduced visual acuity, lid edema—occurred in all analyzed cases. For the detection of etiological agents of keratitis, slit lamp and in vivo confocal microscopy, non-invasive techniques were applied as in earlier studies [3,21,22].

**The laboratory examinations**

The corneal material from affected eyes was tested in our parasitological laboratory for confirmation of *Acanthamoeba* sp. based on cellular level. Corneal samples were assessed using contrast-phase light microscope (Leica, Wetzlar, Germany). To detect pathogenic factors, wet-mount slides and permanent smears (Giemsa, Trichrome) were examined. The identification of trophozoites and cysts of species from the *Acanthamoeba* genus was performed based on their morphology [3,6,21].

**Molecular techniques applied for the species identification of *Acanthamoeba* isolates**

The extraction of DNA from samples of the corneal scrapings was performed in the Department of Tropical Parasitology, Faculty of Health Sciences, Medical University of Gdansk, using the commercial Sherlock AX Kit (A&ABiotechnology, Gdynia, Poland); then, templates of DNA were stored at −20 °C. The genotyping of the keratitis strains using sequence analysis of the 18S rDNA gene was applied. The PCR method was used for specific detection of *Acanthamoeba* DNA, defined according to the procedure by Schroeder et al. [23]. The forward primer JDP1 (5’GGCCCAGATCGTTTACCGTGAA3’) and the reverse primer JDP2 (5’TCTCACAAGCTGCTAGGGAGTCA3’) targeting ~450 bp fragment of *Acanthamoeba* 18S rRNA gene were used. Amplifications according to original previously described conditions in a GeneAmp PCR System 9700 thermocycler were performed (Applied Biosystems, Waltham, MA, USA). PCR products were analyzed using the GelDoc-It Imaging Systems (UVP LLC, Upland, CA, USA) after electrophoresis on agarose gel (Sigma, St. Louis, MO, USA), stained with Midori Green DNA Stain (Nippon Genetics Europe, Duren, Germany).

Direct sequencing was performed using standard procedures and amplification primers; sequences were analyzed using GeneStudio™ Professional (GeneStudio, Inc., Suwanee, GA, USA) and then compared with the sequences available in the GenBank using NCBI BLAST (http://www.ncbi.nlm.nih.gov/BLAST, “accessed on 21 June 2024”.

**Laboratory differential diagnosis**

Corneal samples originating from the cases were tested with the microbiological techniques for species identification in the Department of Pharmaceutical Microbiology, Medical University of Warsaw. Conventional in vitro techniques were preliminarily applied for detection of bacteria and fungi. Microscopic identification of Gram-positive and Gram-negative bacteria strains and in vitro culture techniques were applied for the specific identification of bacteria.

The swab material was grown aerobically on bacteriological agar and on agar with 5% defibrinated sheep blood. Chapman’s plate growth medium was applied for recovery and isolation of *Staphylococcus* strains, McConkey’s medium for Enterobacteriaceae. To isolate fungi, Sabouraud substrate was applied, while for the identification of *Candida* species Chromagar Candida BBL tests was performed.

In vitro cultivation of the samples of isolates was performed for the detection of *Acanthamoeba* sp. under axenic conditions, as in earlier studies, with the use of the Bacto Casitone, in the absence of external food organisms [21,22,26]. Cultures were grown in vitro in sterile 15 mL tubes, with liquid medium composed of Bacto Casitone, Difco (BSC) dissolved in water, enriched with 10% calf serum, at 24 °C, with addition of aqueous solution of antibiotics (streptomycin, penicillin) and sub-cultured into this medium twice a month. *Acanthamoeba* strain’s dynamics parallel cultivated were in vitro monitored and compared to one another; the viability of particular strains, expressed as the ability of trophozoites to multiply, was assessed. Changes in overall numbers of amoebae and cyst/trophozoite proportions, with the aid of the Bürker hemocytometer, were directly counted. Ranges of three counts of the amoebae calculated for 1 mL of the medium were compared for particular strains and assays. Results were analyzed statistically (ANOVA, Student–Newman–Keuls method; the level of statistical significance was set at *p* < 0.05).

## 3. Results

Data of twelve cases of Polish patients with complicated keratitis were evaluated to identify or verify infectious factors and to assess agents that could cause the pathogenic changes in the eyes posing a serious risk for human health. There were various periods in different health units, from eye disease symptom onset to the admission to the Clinics and the *Acanthamoeba* detection, ranging from 23 to 39 days in most cases.

In all incidents investigated, reduced visual acuity appeared in the clinical symptoms with various intensity. Initially, the keratitis cases were treated with antibiotic/antiviral/antifungal medications, with poor results. After *Acanthamoeba* strains had been taken into account as etiological agents of keratitis, the treatment was modified, and involved topical application of chlorhexidine digluconate, propamidine isethionate (Brolene) and polyhexamethylene biguanide (PHMB) with an addition of antibiotics; however, successful treatment has not yet been fully established.

The duration of this anti-*Acanthamoeba* combined therapy and clinical response differed in several cases. Surgical interventions—epithelial debridement, deep anterior lamellar keratoplasty (DALK) or penetrating keratoplasty (PK), amniotic membrane transplantation, and cataract surgery in some severe cases—were needed [6,21,30].

There were risk factors predisposing to *Acanthamoeba* and microbial infections.

From among the 12 complicated keratitis cases investigated, 11 incidents referred to contact lenses having some connection with humans’ exposure to water environments. Probable factors predisposing to AK in some cases was associated with a swimming in the pool, washing in tap water, and in one case a bathing in CL in sea water.

**Figure 1:**
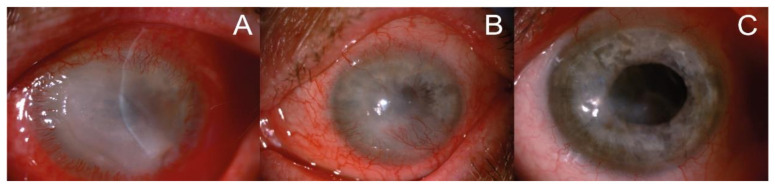
Representative slit lamp images of the patient’s eye affected by *Acanthamoeba* keratitis. (**A**) At the beginning of the treatment: corneal oedema, ring-shaped extensive infiltration covering 2/3 of the corneal surface, severe ciliary congestion and corneal neovascularization; (**B**) after 6 weeks of therapy: significant reduction of corneal edema, decrease in the magnitude of infiltration, more intense corneal neovascularization and improvement in the corneal structure; (**C**) at the end of the treatment: the translucent, stable cornea with regressed vessels. Corneal scar in anterior corneal stroma is covered with healthy epithelium, the anterior chamber is clean, and there are no pathological post-inflammatory vessels in the iris.

The clinical assessment of affected eyes, as in earlier investigations [3,21,22], included non- invasive techniques using the slit lamp and in vivo confocal microscopy. In the slit lamp, pathological changes in affected eyes were observed in the course of severe keratitis; at first, involving the epithelium of the cornea, and then extending to the deeper layers progressively (with pseudo-dendrites, ring infiltrate, corneal ulceration, hypopyon). Visual impairment, congestion of the eyeball, unbearable pain and diffuse irregular epithelial edema progressing to ulcer allowed to take into account *Acanthamoeba* infections; characteristic ring-like stromal infiltrations were found in eight of the cases analyzed. Representative slit lamp images of the eye affected by *Acanthamoeba* sp. at early and advanced stages of AK are presented in Figure 1.

Wet-mount slides directly examined *Acanthamoeba* using a contrast-phase microscope (100× and 400×): the rounded or polygonal in shape, double-walled *Acanthamoeba* cysts (~10–20 μm), (ectocyst, endocyst) with ostioles covered by plugs, so-called opercula; and live, changing in shape trophozoites (~18–40 μm), moving by acanthopodia with spine-like protrusions were detected. The corneal isolates obtained from the affected eyes were identified based on the cellular level as species belonging to the *Acanthamoeba* genus.

In vitro cultivation of corneal isolates undertaken to detect the protists and monitor *Acanthamoeba* viability, with carried out under the axenic conditions with the use Bacto Casitone. In samples of the cultures examined with use of the light microscope, live trophozites moving by acanthopodia and double-walled amoebic cysts were detected based on their morphology.

The cultivation technique is still regarded as the gold standard for the diagnosis of infectious keratitis. Regular evaluation of the culture’s viability expressed as the ability to multiply of particular strains was performed.

The in vitro activity, expressed as intense multiplication for at least eight cycles of sub-culturing were observed in seven incidents. A comparative assessment of in vitro cultured Acanthamoeba corneal strains, as in earlier studies [21,22,26] under the axenic condition in BSC medium, allowed to check the physiological status of the cultivated isolates.

Molecular examinations of the corneal samples cultured in vitro.

Results of the examinations compared with the sequences that were obtained for specific identification revealed 99.3–100% homology, with those available in GenBank identifying as belonging to the T4 genotypes, accession numbers in GenBank MZ401144, MZ401145, MZ401147- MZ401152.

The microbiological examinations of corneal samples performed to determine concomitant infections showed the co-occurrence of protists and microbiota.

In seven cases, *Acanthamoeba* and bacteria occurred; in four cases, *Acanthamoeba* and fungi were revealed. Among the bacteria, Gram-positive strains *Enterococcus faecalis*, Gram-negative *Enterobacter agglomerans*, and *Pseudomonas aeruginosa* co-occurring with amoebae were detected. Simultaneously, the fungi *Fusarium* sp., *Aspergillus* spp. and *Candida* spp. in co-infections with the *Acanthamoeba* occurred. The etiologically mixed keratitis, *Acanthamoeba* sp. and concomitant infectious agents, were identified in ten incidents.

Concomitant potentially infectious factors identified in etiologically complex incidents of *Acanthamoeba* keratitis are presented in Table 1.

In the verification of initial diagnosis, in vivo confocal microscopy was applied. Hyper-reflective double-walled polygonal or round objects, with the outer wall more reflective than the internal one (presumably *Acanthamoeba* cysts) were detected in the corneal epithelial layer.

Concomitant potentially infectious microorganisms were revealed in 9 of 12 *Acanthamoeba* keratitis incidents.

In vivo confocal microscopy scans showed co-occurring fungi and *Acanthamoeba* spp.

**Figure 2:**
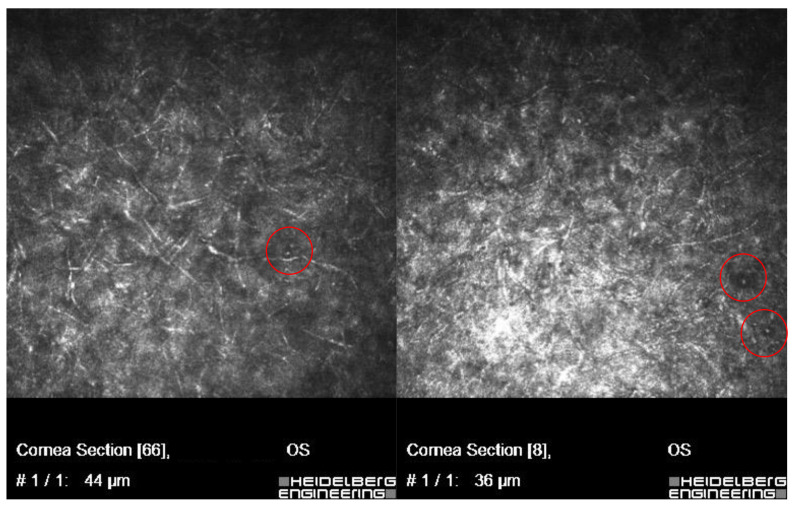
In vivo confocal microscopy scans showing co-infection of *Fusarium* spp. with *Acanthamoeba*.

**Figure 3:**
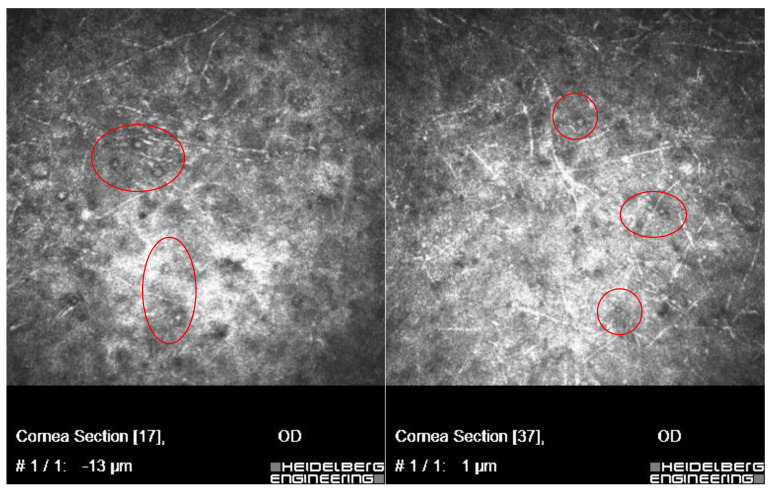
In vivo confocal microscopy scans showing co-infection of *Aspergillus* spp. with *Acanthamoeba* (cysts in red circles).

Figure 2 and Figure 3 present *Fusarium* spp. with *Acanthamoeba culbertsoni* and *Aspergillus* spp. with *Acanthamoeba* sp.

**Figure 4:**
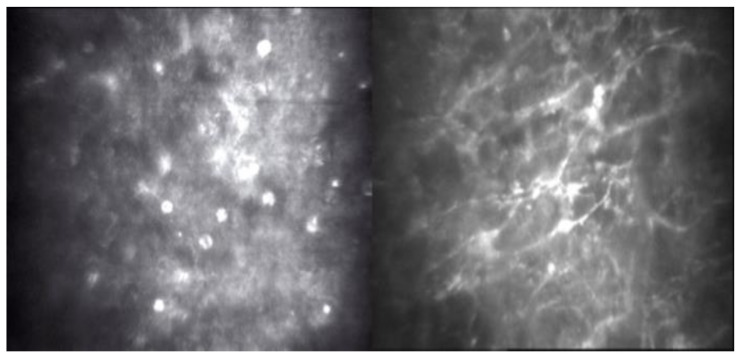
In vivo confocal microscopy scans showing co-infection of filamentous fungi with *Acanthamoeba*.

Figure 4 presents *Acanthamoeba* sp. and some unidentified species of the dimorphic fungi converted to filamentous form. In these three cases, risk factors predisposing to *Acanthamoeba* and concomitant infections concerned wearing contact lenses (CL).

**Figure 5:**
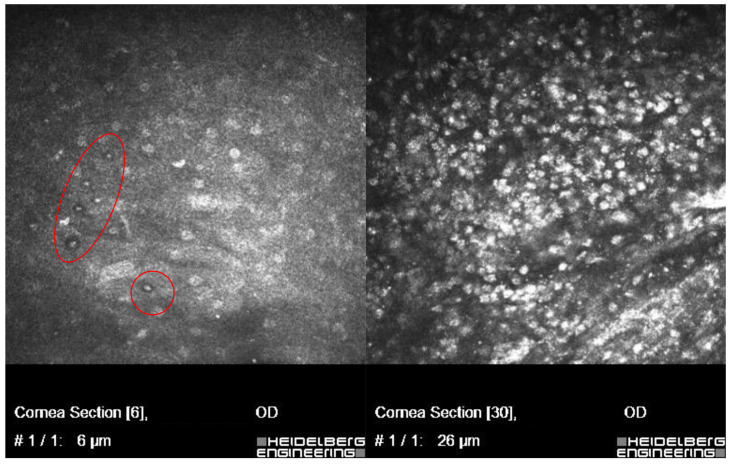
In vivo confocal microscopy scans showing co-infection of bacteria *Pseudomonas aeruginosa* and *Acanthamoeba* (cysts shown in the left side of the picture). Right side of the picture shows massive inflammatory response.

In vivo confocal microscopy scans showed co-occurring bacteria *Pseudomonas aeruginosa* with *Acanthamoeba*; the scans are presented in Figure 5.

In one other severe case of keratitis, in which the in vivo confocal microscopy has been used, unexpectedly, actively motile worms that have morphological features of the Filarioidea, in co-infection with *Acanthamoeba* sp. were found.

**Figure 6:**
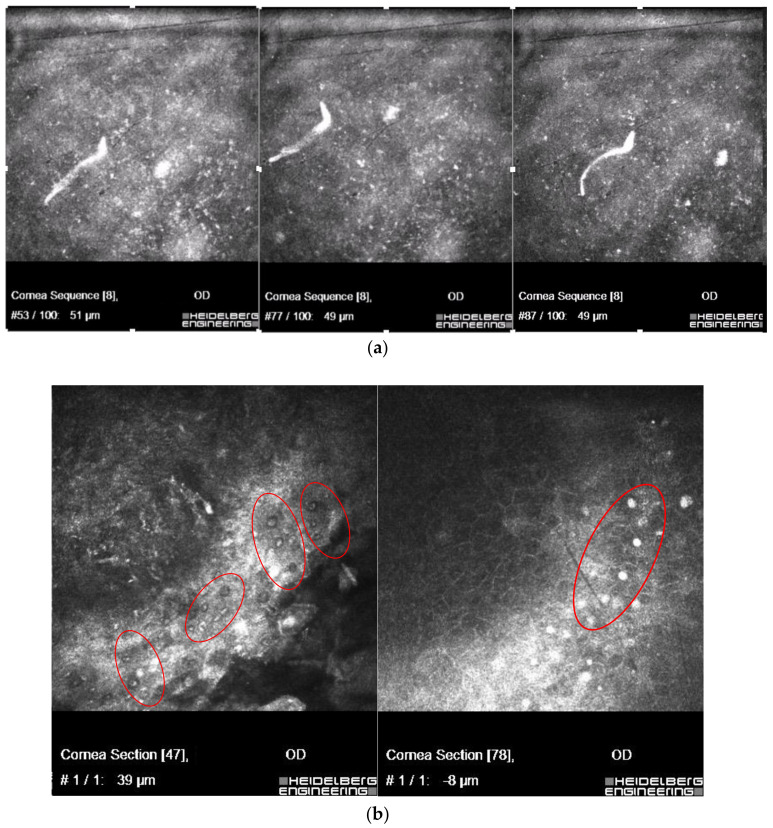
In vivo confocal microscopy scans showing co-infection of live microfilaria (**a**), with *Acanthamoeba* (in red circles) (**b**).

The patient was a 30-year-old woman, a sailor on cruise, who was bitten by flies, and probably bathed in sea water while using contact lenses. Three weeks later, the patient presented with eye symptoms (keratitis) and general symptoms (fever, musculoskeletal pain). For 3 days, Amoxiclav and Ibuprofen were implemented—nothing more was available on the cruise. A week later, on ophthalmologist con-sultation, moxifloxacine, ofloxacine and dexamethasone were implemented, without improvement. After re-examination by confocal microscopy, a strong suspicion of *Acanthamoeba* keratitis was revealed and, probably, co-infection with *Onchocerca* sp. The corneal scrapings were taken for direct examination. The treatment was immediately modified: 0.2% chlorhexidine and 0.1% propamidine were added. Material was re-collected after one week; no Dirofilariae were found.he patient with keratitis had earlier been bitten by flies; the parasite was found to be concomitant with *Acanthamoeba* sp. (identified as *Onchocerca* sp., (Nematoda) with Filarioidea features - with unsheathed microfilariae, distributed by *Simulium* blackfly in specific endemic areas in Brazil. In vivo confocal microscopy scans showing co-infection of live microfilaria with hyper-reflective double-walled *Acanthamoeba* cysts are presented in Figure 6a,b.

## 4. Discussion

The serious health threat caused by pathogenic strains of amphizoic amoebae is an emerging public problem worldwide [14,18,23,24,25,26,27,28,29,30,31,32,33]. *Acanthamoeba* spp. existing as free-living heterotrophs as well as facultative parasites, can cause serious medical risk.

*Acanthamoeba* keratitis (AK) that easily leads to blindness is caused by pathogenic strains of the amphizoic amoebae. AK caused by the amoebae is also a growing human health threat in Poland [22,26,34,35,36,37,38,39,40].

These protists are found in diverse climate regions in various environments where they are ubiquitous in natural and man-made aquatic environment [6,11,19,29,30,31,32,33,34,35,36].

Identification of environmental and clinical isolates of the amoebae examined on a cellular level with the use of culture methods is based on morphological characteristics. *Acanthamoeba* molecular methods of classification is based on genotype associations in the 18S rRNA gene sequence, which divides the genus into 20 different evolutionary lines or clades.

*Acanthamoeba* keratitis is a sight-threatening corneal disease that is caused by at least eight species of *Acanthamoeba*: *A. castellanii*, *A. culbertsoni*, *A. polyphaga*, *A. hatchetti*, *A. rhysoides*, *A. lugdunesis*, *A. quina*, and *A. griffini* [21,37].

A pathogenesis of *Acanthamoeba* keratitis is a multi-factorial process.

Adhesion of the *Acanthamoeba* trophozoites to host cells followed by phagocytosis to take up food particles are factors contributing directly.

The proportion of trophozoites and cysts dependent on the environmental conditions, the ability to exist in different environments and to adapt to various human organs and tissues, including the human cornea, are believed to be factors contributing indirectly [21,22].

The keratitis caused by amphizoic amoebae in humans poses challenges in terms of, among others, prevention, differential diagnosis, risk factors and treatment; all of which occurred in our study.

The ability to prevent the spread of *Acanthamoeba* is limited due to the remarkably wide range of habitats where amphizoic amoebae commonly occur.

Non-specific symptoms of AK that are similar to those observed in the course of microbial keratitis, not accompanied by unequivocal pathognomonic signs, may cause serious difficulties in differential diagnosis, delay proper therapy and result in serious infection.

Since the specific amoebicidal agents are not available, the treatment of AK still poses a problem.

The resistance to initially applied antimicrobial therapy appeared in the cases assessed in our study. The *Acanthamoeba* pathogenic strains detected in the incidents in our research, and the resistance to applied therapy observed in the cases were taken into account as the important criterion for their inclusion to our study. It is known that differential diagnosis and treatment of AK is challenging, due to the extreme resistance of amoeba cysts to disinfectants; anti-microbial anti-parasitic drugs often are diagnosed at a late stage [3,6,21,22,28].

In this interdisciplinary study, based on the literature data and our experience, incidents with suspicion of infectious keratitis were evaluated in aspects of risk factors for AK, the possibility of determination of the pathogenic amoeba strains, the detection of concomitant infections and response to applied therapy [8,9,10,11,12,13,15,16,17,18,19,28,30].

Because some mistakes were made in the identification of causative agents of keratitis and ineffective treatment that occurred initially in most cases, diagnostic verification was needed. Following the results of laboratory investigations, the treatment was modified, and triple-topical therapy (polyhexamethilen-biguanide, propamidine-isethionate, neomycin) was applied. In some severe incidents, surgical interventions were needed [6,21,30].

Our study confirmed current data and provided further information on the clear connection of serious risk of *Acanthamoeba* spp. infections with wearing contact lenses and exposure to water habitats.

In 11 of 12 incidents assessed, the highest risk of AK was connected with wearing contact lens (CL) associated with water environments: wearing contact lenses while swimming in the pool, washing CL in tap water and in one case bathing in CL in sea water [9,10,11,12,13,14,21,22,27].

Microbial keratitis caused by the *Pseudomonas aeruginosa* is commonly associated with contact lens wear in the connection in some water habitats; contact lens wearing is the main factor predisposing to the *Acanthamoeba* and concomitant infections. Special emphasis should be given to the assessment of the circumstances predisposing to the development of the *Acanthamoeba* infections and co-occurring microbial eye diseases, as well as the effect of associated factors [6,15,17,19,36].

The microbiological examinations of corneal samples showed the *Acanthamoeba* trophozoites and cysts inside the eye. Simultaneously, valuable data on co-occurring live *Acanthamoeba* developmental forms, and bacteria and fungi were received by in vivo confocal microscope.

The concomitant *Acanthamoeba* pathogenic strains detected in the incidents assessed in the study were: *Acanthamoeba* sp., *A. castellanii*, *A. mauritanensis* and *A. culbertsoni* and co-occurring bacteria or fungi: *P. aeruginosa*, *E. faecalis*, *Fusarium* sp., *Candida* spp., *Aspergillus* spp., dimorphic fungi in filamentous form converting to yeast form. All strains were revealed in these etiologically complex keratitis cases.

The microorganisms are potential factors posing serious health risks, particularly dangerous for people with impaired immune systems.

The free-living amoebae are environmental protists that contribute to microbiological contamination of water sources, being at the same time the vector for pathogens. The amoebae may transmit endosymbionts that are potentially pathogenic for humans and are able to survive/proliferate intracellularly within the amoebae [32,33,34,35,36,37,38]. It is considered that endosymbionts from *Acanthamoeba* trophozoites and *Pseudomonas* sp., can influence the pathogenicity/resistance of *Acanthamoeba* strains to terapeutic agents [25,26,27,28,29,30,31,32,33,34,35].

Our research confirmed the literature data that *Acanthamoeba* strains are found in diverse climatic regions and ecological habitats; they are ubiquitous in natural/man-made aquatic environment [6,11,19,29,30,31,32,33,34,35,36].

In this study, in severe keratitis incident, motile microfilariae co-infected with *Acanthamoeba* sp. were detected by confocal microscopy. The worms detected in our investigations of the keratitis cases has been identified as *Onchocerca* sp. (Nematoda) with morphological features of Filarioidea, with unsheathed microfilariae, distributed by *Simulium* blackfly in specific endemic areas; e.g., Brazil. Two developmental forms: adults and microfilariae occur in life cycle of the round-worms. In accordance with literature, the ocular diseases caused by the zoonotic or man species of Filarial nematodes, transmitted by the arthropod vectors to the vertebrate hosts are reported to be more common in regions with favorable (environmental, epidemiological) factors [41,42,43,44,45,46]. To the best of our knowledge, this is the first case of detection in Poland of an eye infected by microfilariae of *Onchocerca* sp. as a concomitant factor co-occurring with *Acanthamoeba* infection.

## 5. Conclusions

The corneal samples originating from the affected eyes were assessed in the study to identify/verify infectious factors that could cause pathogenic changes in the eyes and thus constitute a serious risk to human health.

The complex clinical picture and interrelations occurring between the *Acanthamoeba* pathogenic strains and concomitant bacterial and fungal factors identified in keratitis at cellular and molecular levels influenced disease management.

Diagnostic difficulties/misdiagnoses and earlier ineffective AK treatment forced us to verify infectious factors. Direct examinations of corneal scrapings confirmed/verified the diagnosis by detection of the live protozoans. Non-invasive techniques with the use of slit lamp and in vivo confocal microscopy performed in terms to determine concomitant infections showed the co-occurrence of protists and microbiota.

In cases of the contact lens-related keratitis, the long time from symptom onset to identifying etiological factors can impact the complicated course of management.

Results of the study showed that *Acanthamoeba* infection should be early suspected, although no risk factor predisposing to AK was identified. Greater awareness of the medical importance of preventive actions is useful to inhibit the spread of *Acanthamoeba* infections.

If no or a weak response to the applied therapy occurs, a risk of the *Acanthamoeba* spp. infections with simultaneous potentially contagious factors should be taken into account.

In the assessment of agents affecting the complex course of the disease, applying combined treatment, and early identification of concomitant factors/pathogens are necessary.

Currently, awareness and knowledge about AK—the serious, vision-threatening eye disease are still insufficient; an improvement in duration from first symptoms appearance to suitable diagnosis is urgently needed.

## Figures and Tables

**Table 1:** Concomitant infectious factors identified in etiologically complex *Acanthamoeba* keratitis.

Corneal Samples		Accession No in GenBank	Concomitant Infection’s Agent	Time from Symptom Onset to *Acanthamoeba* Detection
CS2f	*Acanthamoeba* sp. #	-	*Citrobacter* sp. *Candida* spp.	32 days
CS 3f	*A. castellanii* ~	MZ401144	*P. aeruginosa*	26 days
CS 4f	*A. castellanii* #	MZ401145	*P. aeruginosa*	6 days
CS 6f	*A. mauritanensis* >	MZ401147	not detected	32 days
CS 8m	*A. castellanii* ^	MZ401150	*P. aeruginosa*	28 days
CS 9m	*A. castellanii* #	MZ401151	*E. faecalis*	39 days
CS10f	*Acanthamoeba* sp. #	MZ401148	*Candida* spp.	116 days
CS 11f	*A. culbertsoni* #	MZ401149	*P. aeruginosa* *Fusarium* sp.	12 days
CS13m	*A. castellanii* #	MZ401152	not detected	26 days
CS 23m	*Acanthamoeba* sp. #	-	*P. aeruginosa* *E. cloacae*	23 days
CS 31f	*Acanthamoeba* sp. *	-	Microfilaria	34 days
CS 34f	*Acanthamoeba* sp. #	-	*Aspergillus* spp.	28 days

Probable factors predisposing to AK: # CL wearing, > washing in CL in tap water, ^ swimming in CL in a pool, * bathing in CL in sea water, ~ not identified, not wearing CL.

## Data Availability

The data are not publicly available due to patient privacy and ethical reasons.
